# Opportunities and Challenges for Single-Unit Recordings from Enteric Neurons in Awake Animals

**DOI:** 10.3390/mi9090428

**Published:** 2018-08-25

**Authors:** Bradley B. Barth, Hsin-I Huang, Gianna E. Hammer, Xiling Shen

**Affiliations:** 1 Department of Biomedical Engineering, Duke University, Durham, NC 27710, USA; xiling.shen@duke.edu; 2 Department of Immunology, Duke University, Durham, NC 27710, USA; hsin.i.huang@duke.edu (H.-I.H.); gianna.hammer@duke.edu (G.E.H.)

**Keywords:** microelectrodes, in vivo electrophysiology, neural interfaces, enteric nervous system, conscious recording, electrode implantation

## Abstract

Advanced electrode designs have made single-unit neural recordings commonplace in modern neuroscience research. However, single-unit resolution remains out of reach for the intrinsic neurons of the gastrointestinal system. Single-unit recordings of the enteric (gut) nervous system have been conducted in anesthetized animal models and excised tissue, but there is a large physiological gap between awake and anesthetized animals, particularly for the enteric nervous system. Here, we describe the opportunity for advancing enteric neuroscience offered by single-unit recording capabilities in awake animals. We highlight the primary challenges to microelectrodes in the gastrointestinal system including structural, physiological, and signal quality challenges, and we provide design criteria recommendations for enteric microelectrodes.

## 1. Introduction

**Figure 1:**
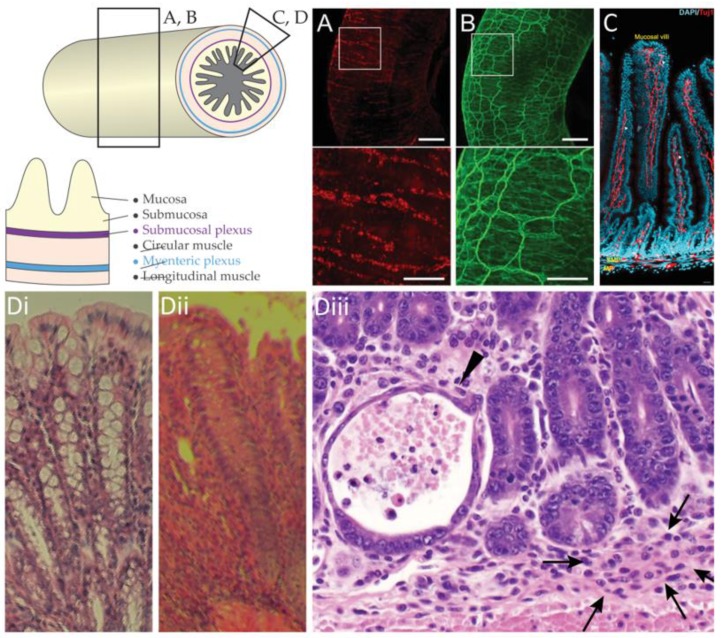
Anatomy of the enteric nervous system. A segment of the gastrointestinal tract and the anatomical tissue layers. Pan-neuronal marker HuC/D (**A**) and neuron tubulin marker Tuj-1 (**B**) imaged in whole intestinal tissue by light sheet microscopy, adapted from [6]; (**C**) Immunoreactive labelling of cell nuclei (DAPI, blue) and neuron tubulin (Tuj-1, red) in sections of the intestine, adapted from [7]; (**D**) Histology of (**i**) healthy colon; (**ii**) inflamed colon; and (**iii**) inflamed small intestine with crypt abscess (arrowhead) and granuloma (arrows).

The enteric nervous system is a subdivision of the peripheral, autonomic nervous system that resides in the gastrointestinal tract (Figure 1A–C). The small intestine alone has been estimated to contain more than 733,000 neurons in the mouse, 3.7 million neurons in the guinea-pig, and 88 million neurons in the sheep [1]. The human enteric nervous system is estimated to contain between 200 and 600 million neurons, roughly as many as the spinal cord [2]. For over a century, the enteric nervous system has been known to regulate gastrointestinal motility, and the circuitry controlling basic motor patterns is relatively well understood [3]. Pathologies of the enteric nervous system include functional and motility disorders, developmental disorders, and neurological disorders [4,5].

Despite its size and importance, the enteric nervous system is under-examined compared to other systems in neuroscience. Our knowledge of enteric neuroscience remains antiquated compared to the central nervous system because of the lack of specialized tools and methods. For instance, it has been possible to record cortical neurons intracellularly in freely-moving animals [8], and calcium activity from populations of cortical neurons in head-fixed animals [9] for over a decade. In contrast, recordings from the enteric neurons have been conducted almost exclusively in excised tissue.

Classical enteric electrophysiology is conducted using flat-sheet preparations, a method that has remained largely unchanged for decades. As enteric neuroscience progresses, flat-sheet preparations are not sufficient to investigate the interactions of the enteric nervous system with other systems, including the gut-brain axis, neuro-immune crosstalk, interaction with microbiota, etc., in living systems. For proper context, our understanding of these systems will be enhanced by measurements in live animal models, which offer greater physiological fidelity and greater potential for translational research. However, technology for awake, single-unit recordings in the gastrointestinal system is underdeveloped.

Currently, in vivo neural recordings from the gastrointestinal tract must be conducted under anesthesia, presumably during acute, non-survival surgical procedures. Anesthesia and invasive surgical procedures greatly alter the physiology of the gastrointestinal environment, directly affecting neurotransmission and motility. To fully realize the advantages of in vivo enteric electrophysiology, neural recording and stimulation must be conducted in conscious animal models. Advancing neurogastroenterology with the tools for single-unit recordings in awake animal models demands new and innovative neural microelectrode technology.

First, we review the traditional methods for enteric electrophysiology, discussing ex vivo preparations and the limitations of anesthetized in vivo neural recordings. Secondly, we discuss the current challenges to single-unit recordings from enteric neurons in awake animal models, such as gastrointestinal pathophysiology (Figure 1D). Finally, we consider design criteria for novel enteric microelectrodes and potential applications of single-unit recordings from conscious animals and the potential synergy with other novel technologies.

## 2. Classical Methods for Enteric Electrophysiology

**Figure 2:**
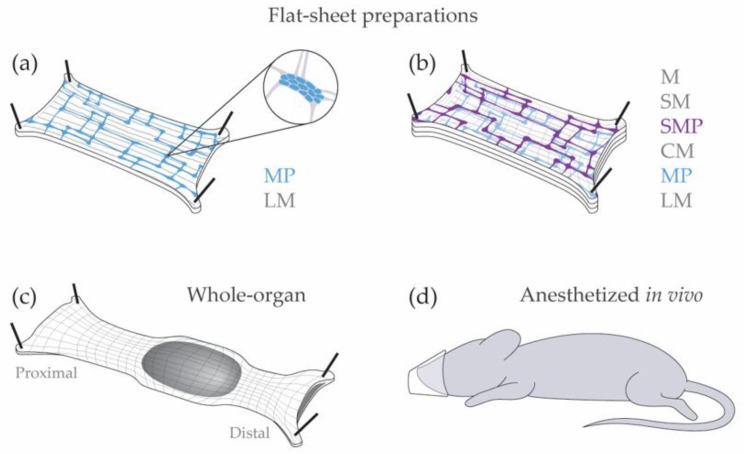
Classical methods for enteric electrophysiology. (**a**) Flat-sheet LMMP preparation; (**b**) Full-thickness flat-sheet preparation; (**c**) Whole-organ preparation; (**d**) Anesthetized in vivo preparation. M: mucosa, SM: submucosa, SMP: submucosal plexus, CM: circular muscle, MP: myenteric plexus, LM: longitudinal muscle.

Electrophysiology in the enteric nervous system has largely been conducted in excised tissue (Figure 2). Excised tissue can be kept alive and functional for several hours, often with direct access to enteric ganglia. More complex preparations have been developed to capture neural activity with greater physiological relevance, such as suction electrodes for whole-organ recordings. Enteric neuron recordings are rarely conducted in vivo. In this section, we discuss the advantages and limitations of flat-sheet and whole-organ preparations, and the challenges of anesthetized recordings.

### 2.1. Neural Recordings in Excised Tissue

Enteric neural recordings are most commonly conducted *ex vivo*, using flat-sheet preparations in organ baths. In these preparations, the gastrointestinal tract is dissected out, opened along the mesenteric border, and pinned flat in a Sylgard dish. The mucosa, submucosa, and circular muscle is frequently dissected away, leaving only the myenteric plexus attached to the longitudinal muscle (LMMP) [10]. The flat-sheet LMMP preparation was fundamental for the intracellular recordings that first classified electrophysiology in enteric neurons as S (Type 1) or AH (Type 2) neurons [11,12]. Although the electrophysiology classification system is less frequently used than neurochemical or functional classification [13,14], it is often used to characterize patient biopsies [15]. The primary advantage of this preparation is the accessibility of myenteric ganglia for pharmacological assays with extracellular recordings, patch clamp recordings, etc. [16]. However, the flat-sheet LMMP preparation has limited applications because the submucosal plexus, circular muscle, lamina propria, and epithelium have been dissected away. Therefore, this preparation is not suitable for examining the effect of intraluminal stimuli or communication with epithelial cells, resident immune cells, submucosal neurons, or circular muscle. 

Alternatively, the full-thickness flat-sheet preparation maintains the connections to circular muscle, submucosal plexus, lamina propria, and epithelium. As a result, the full-thickness flat-sheet preparation is ideal for examining intraluminal stimuli and interactions between enteric neurons and the epithelium, resident immune cells, and smooth muscle. For example, Spencer and colleagues have revealed novel firing patterns in enteric neurons that drive coordinated smooth muscle response using the full-thickness flat-sheet preparations [17,18]. The full-thickness flat-sheet preparation is also advantageous for calcium imaging because it captures either plexus in a single imaging plane [19,20]. However, myenteric and submucosal neurons are enclosed within the smooth muscle layers and the lamina propria in the full-thickness flat preparation, making single-unit and intracellular recordings prohibitive in this preparation. A fundamental limitation of all flat-sheet preparations is the longitudinal incision along the mesenteric border. This incision disrupts the electrical syncytium, particularly in the circular muscle, and severs many circumferentially projecting fibers. Further, the flat-sheet preparation is not well equipped to propel luminal contents.

Gastrointestinal motility patterns are better examined in whole-organ preparations [21,22]. Whole-organ preparations maintain the intrinsic connections of the enteric nervous system, leaving the smooth muscle, lamina propria, and epithelial layers intact. Whole-organ preparations consist of intact segments of the gastrointestinal tract in organ baths, and they are well-suited for examining gastrointestinal motility patterns or intraluminal stimuli because the longitudinal and circular smooth muscles remain functional and intact. As with the full-thickness flat-sheet preparation, the enteric neurons in whole-organ preparations are inaccessible by classical electrophysiology methods. Suction electrodes on the serosal surface provide an alternate method by measuring smooth muscle activity in whole-organ and full-thickness flat-sheet preparations, but they are inadequate to describe enteric neural activity directly [23,24,25].

Neural recordings from excised tissue present a convenient platform for examining single-unit response under a variety of conditions and stimuli. However, several limitations exist for all excised tissue preparations, including, most notably, the lack of peripheral innervation and extrinsic circuitry. In some ex vivo preparations, peripheral fiber recordings are possible, but they lack extrinsic circuits in the central nervous system [26,27]. The limitations of ex vivo preparations can be addressed by studying the enteric nervous system in live animal models.

### 2.2. Challenges of Anesthetized Recordings from Enteric Neurons

Anesthesia allows for recordings from live animal models, which provide more physiologically-relevant conditions compared to excised tissue. Due to current technological limitations, flat-sheet preparations are better suited for single-unit recordings than anesthetized recordings. Additionally, anesthesia greatly changes gastrointestinal function, making results from anesthetized preparations difficult to interpret. We discuss two direct effects of various anesthetic agents on gastrointestinal function: the effect of anesthesia on various receptors of the enteric nervous system, and the effect of anesthesia on gastrointestinal motility.

First, several neuron species in the enteric nervous system act on receptors that are directly affected by various anesthetic agents. Here, we review the inhibiting and potentiating effects of common anesthetic agents on some of the primary receptor classes in the enteric nervous system: nicotinic cholinergic, P2X, 5-HT_3_, N-methyl-D-aspartate (NMDA), α-amino-3-hydroxy-5-methyl-4-isoxazolepropionic acid (AMPA), gamma-Aminobutyric acid (GABA), and glycine receptors (Table 1). Agonists to these receptors are expressed by common neuron species in the myenteric ganglia and submucosal ganglia [13,28,29,30]. Although glutamate and glycine are less well-studied in enteric ganglia in comparison to acetylcholine, serotonin, and purinergic neurotransmitters, their role as enteric neurotransmitters are strongly supported by electrophysiological responses to pharmaceutical stimuli [30,31]. The receptor-specific responses for several forms of anesthesia have been reviewed by [32]. In addition to the direct effects of anesthesia, [33] have reported that common anesthetic agents (isoflurane, sevoflurane, ketamine, and urethane) modulate glutamate receptors, voltage-dependent calcium channels, and voltage-gated potassium channels, suggesting that anesthesia may have prolonged effects on neural activity.

Secondly, commonly used anesthetic agents impair gastrointestinal motility. Here, we review the effects of commonly used injected and inhaled anesthetic agents (ketamine, urethane, pentobarbital, propofol, isoflurane, sevoflurane, and halothane) on gastrointestinal motility during anesthesia (Table 2). Generally, anesthetic agents have been shown to impair gastrointestinal motility by delaying gastric emptying or decreasing intestinal transit time.

In addition to the effects of anesthesia, invasive abdominal surgery has been shown to impair gastrointestinal motility. For example, human patients who have undergone laparotomy often experience motility disorders such as postoperative ileus or pseudo-obstruction [60,61]. In horses, surgery has been shown to disrupt gastrointestinal motility for 8 to 12 h [62]. Furthermore, complications during surgery can lead to acute acidosis, which has been shown to directly reduce gastrointestinal motility [63]. To mitigate the adverse effects of invasive surgery on gastrointestinal function, animals should be allowed to recover prior to neural recordings or other experiments.

In summary, the flat-sheet preparation is a fundamental tool for enteric electrophysiology, and it will not be replaced by new technology. However, the versatility of ex vivo preparations are limited, and they lack the necessary context to examine more physiologically complex behaviors. Although anesthetized, in vivo animal models are more physiologically relevant, however, anesthesia and invasive surgery alter neurotransmission and impede gastrointestinal motility. Therefore, the effect of various anesthetic agents and sufficient recovery time should be considered in the design of experiments. Importantly, this demonstrates the potential advantages of conducting neural recordings in conscious animals, particularly for neurogastroenterology.

## 3. Challenges to Gastrointestinal Neuro-Electrophysiology in Conscious Animals

Recently, new technology has been developed for myo-electrophysiology in the gastrointestinal system of anesthetized animals and patients. L. K. Cheng and collaborators at the University of Auckland examine smooth muscle function and electrical slow wave, using methods originally developed by [82]. Arrays featuring multiple surface electrodes can be used to build spatiotemporal maps of slow wave propagation with high resolution in anesthetized animal models [83] and in patients during surgery [84]. In vivo myo-electrophysiology has led to an improved understanding of electrical slow wave activity in healthy and diseased models. Although high-resolution myo-electrophysiology has not yet reached conscious animals, it shows great promise, particularly for improved diagnosis of gut pathophysiology. Simultaneously, in vivo gastrointestinal neuro-electrophysiology remains largely out of reach, especially in awake animals. There are several barriers to in vivo gastrointestinal neuro-electrophysiology, most of which are not unique to the gastrointestinal environment, such as fibrosis and biofouling. In this section, we focus on the challenges that are greatly exacerbated in the gut.

We identified six key challenges to in vivo gastrointestinal neuro-electrophysiology across three categories: structural, physiological, and signal quality challenges (Table 3). The structural challenge is the movement of the gastrointestinal tract within the abdomen, worsened by the lack of accessible skeletal structures on which to mount a device. The two physiological challenges describe the risks of disrupting gastrointestinal function: the issue of ischemia and reperfusion, and maintaining gastrointestinal homeostasis. The three signal quality challenges are contamination from the electrical slow wave, smooth muscle action potentials, and artifact due to tissue movement.

### 3.1. Structural Challenges in Neurogastroenterology

Animal movement is problematic for all methods of awake electrophysiology; movement adds noise to the recording, damages the recording device, and harms the test subject. Generally, the effect of conscious movements on neural recordings can be mitigated in two ways: restraining the animal, such as head-fixed recordings, or minimizing aberrations in movement by fixing the recording device to the skeleton. Restrained recordings pose fewer movement-related problems than unrestrained (a.k.a. freely-behaving) recordings, but the restraint method may alter natural neural activity. For example, single-unit recordings from freely-moving rats led to the discovery of place cells in the hippocampus [85]. These methods have proven useful tools for probing the brain, and are adaptable for other systems; head-fixed preparations, for example, have led to spine-fixed recordings and spinal recordings in awake, moving rats [86,87]. However, these advancements have not led to similar innovation in enteric neuroscience because of unique movement-related challenges posed by the gastrointestinal environment.

Awake, single-unit recordings from enteric neurons are limited by structural challenges in the gastrointestinal system. First, there are no accessibly skeletal structures below the stomach on which to mount rigid devices, as used in brain and spine research. Additionally, enteric neurons are not fixed in place within the abdominal cavity. Enteric neurons are located within the wall of the gastrointestinal tract. In the gastrointestinal wall, smooth muscles drive macroscopic tissue motion in the form of stationary or propagating waves of contractions, known as segmentation and peristalsis, respectively. Smooth muscle contractions can induce tissue displacement several orders of magnitude greater than micromotions observed in the brain. For example, micromotions in the brain have been observed on the order of 10 to 100 µm in rats [88]. Meanwhile, maximum distension in the colon can deform the circular muscle up to 10 mm in guinea-pigs [89].

Movement-related challenges are amplified in the gastrointestinal system. Future implantable devices must consider the mechanical characteristics at the tissue, organ, and body scales. Such devices will likely combine flexible electrode arrays and interconnects, and rigid headstages mounted far from the recording site. Additionally, the inflammation and irritation caused by sutures or adhesives must be considered.

### 3.2. Disrupting Gastrointestinal Physiology

The gastrointestinal tract has evolved defense mechanisms that pose significant challenges for medical device implants, particularly neural microelectrodes. In addition to the foreign-body response associated with all medical implants, the gastrointestinal system poses unique challenges. Here, we discuss the general principles of maintaining homeostasis in the gastrointestinal tract and the potential challenges of intestinal injury caused by implanting neural microelectrodes. Intestinal injury and inflammation induced by resident immune responses and ischemia reperfusion injury pose challenges for enteric in vivo neuro-electrophysiology because they greatly alter the behavior of enteric neurons, enteric glial cells, and resident immune cells, and disrupt gastrointestinal function.

The mammalian intestine encounters trillions of innocuous foreign antigens, symbiotic microbes, and pathogens daily. The intestinal immune system is able to tolerate innocuous antigens and simultaneously respond to pathogens using three layers of regulation: physical barriers, antimicrobial reagents, and immune cells [90]. First, the intestine is covered by a single lining of intestinal epithelium cells, and specialized intestinal epithelium cells secrete mucus to protect the epithelium from microbiota [91,92]. Second, specialized intestinal epithelium cells also release antimicrobial compounds. For example, Paneth cells express antimicrobial peptides such as RegIIIγ and α-defensin to inhibit luminal microbe growth and colonization in intestine [93]. Third, antigen-presenting cells, including dendritic cells and macrophages, are responsible for immune surveillance and maintaining homeostasis. Intestinal dendritic cells make up the most complex dendritic cell populations in the body, and they are essential for establishing tolerance in the homeostatic environment by promoting regulatory T cells [94,95]. Gastrointestinal macrophages are unique; unlike most tissue-resident macrophages, which are yolk sac or embryo derived with self-renewal capacity, gastrointestinal macrophages are continuously replenished by circulating monocytes and are exquisitely sensitive to environmental stimuli [96,97]. Mature gastrointestinal macrophages maintain epithelial cell integrity, and limit bacteria-induced inflammatory responses by constantly secreting inhibitory cytokines and low levels of tumor necrosis factor (TNF), and engulfing penetrating bacteria via efficient phagocytosis, respectively [98,99]. The intestinal immune system carefully titrates the inflammatory response to innocuous antigens, symbiotic microbes, and pathogens, but it may be dysregulated by implanted neural microelectrodes.

Implanted neural microelectrodes in the intestine have the potential to cause severe intestinal inflammation by disrupting epithelial barrier function and activating antigen-presenting cells. First, epithelial barrier function is importance for homeostasis, and has been implicated in inflammatory bowel disease patients [100,101]. Breaking down epithelial cells in animal models, such as with dextran sulfate sodium or 2,4,6-trinitrobenzenesulfonic acid, has been shown to induce severe colitis and intestinal inflammation [102,103,104,105]. Barrier function can also be disrupted by ischemia reperfusion injury, a common gastrointestinal disease in which hypoxia-ischemia and reperfusion in the epithelium leads to epithelial cell death caused by enhanced reactive oxygen species production once blood flow is re-established in hypoxic regions [106,107]. Disrupted barrier function can lead to bacteria translocation and directly activate enteric neurons and glial cells that express innate pattern recognition receptors, such as toll-like receptors [108,109].

Additionally, intestinal inflammation may be induced by antigen-presenting cells in response to pathogens, translocated bacteria, or when they are dysregulated. For example, intestinal inflammation developed spontaneously in mice after knocking out A20, a nuclear factor kappa-light-chain-enhancer of activated B cells (NF-kB) signaling pathway inhibitor [110]. Distinct dendritic cells, pro-inflammatory monocytes, and pro-inflammatory macrophages promote the intestinal inflammation response, increase differentiation of pro-inflammatory monocytes and macrophages, and production of pro-inflammatory cytokines [111,112,113,114]. Chronic inflammation can mediate enteric neuron cell death, posing additional challenges to in vivo neuro-electrophysiology [115]. Neural microelectrode implants have the potential to disrupt homeostasis and barrier function, induce cell death and bacteria translocation, and lead to chronic inflammation.

### 3.3. Signal Quality

The signal-to-noise ratio of enteric neuro-electrophysiology will likely be contaminated by three main sources of noise specific to the gastrointestinal tract. First, electrical slow waves will introduce low-frequency noise. Second, action potentials from surrounding smooth muscle tissue will contribute high-frequency noise. Third, peristalsis and segmentation will create motion artifact, introducing additional high-frequency noise.

Electrical slow waves propagate through smooth muscle along the length of the gut, from esophagus to rectum, and they are driven by pacemaker cells known as interstitial cells of Cajal [116]. Populations of interstitial cells of Cajal vary along the length of the gut and occupy the myenteric, intramuscular, and submucosal layers and have individual pacemaker frequencies [117]. The pacemaker potentials conduct through the smooth muscle syncytium, generating electrical slow waves [118]. The smooth muscle layers directly border the myenteric and submucosal plexuses, and any recording from the plexus layers will contain signals from electrical slow waves [119]. The slow waves will contribute low-frequency noise, because they occur at 2–40 cycles per minute, depending on animal species and location along the gastrointestinal tract [29]. Therefore, high-pass filtering will remove most slow-wave noise from neural recordings.

Smooth muscle action potentials and motion artifact will contribute physiological noise to neural recordings at high frequencies. Smooth muscle fibers border the myenteric and submucosal plexuses, and recordings from the plexus layers will likely contain neural action potentials and muscle action potentials [120]. For single-unit recordings, it will be difficult to filter out muscle action potentials and claim with certainty that the spiking signals are of neural origin. Extracellular action potential shape analysis or template matching will likely be the most effective way to differentiate these signals [121].

Coincident with smooth muscle activity are macroscopic movements in gastrointestinal tissue, causing artifacts in electrical recordings. Motion artifact is a long-standing issue for gastrointestinal electrophysiology in excised tissue, and it continues to pose challenges for understanding electrical slow waves and characterizing smooth muscle action potentials [122,123]. In classical neuro-electrophysiology in excised tissue, slow waves, smooth muscle action potentials, and motion artifact can be blocked pharmacologically [15]. However, these sources of noise cannot be blocked during in vivo neuro-electrophysiology without disrupting gastrointestinal physiology. Instead, limiting these sources of noise during in vivo neural recordings may be achieved by improved implant design and various signal processing techniques.

## 4. Enteric Microelectrode Design Criteria

The gastrointestinal environment poses unique challenges that have slowed progress in enteric neuroscience. Novel neural microelectrodes designed specifically for the gut may overcome these unique challenges and provide access to single-unit activity for the first time. In this section, we suggest design criteria for enteric microelectrodes for awake, single-unit recordings. The design criteria target the six key challenges to in vivo gastrointestinal neuro-electrophysiology by focusing on: intrinsic material properties, extrinsic design parameters, and the implant procedure (Table 4).

### 4.1. Intrinsic Material Properties

The gastrointestinal tract has high elasticity, and enteric microelectrodes will need to withstand large tissue displacements and strain without failure. Gastrointestinal tissues have an isotropic elastic modulus ranging from 0.3 kPa to 5 MPa depending on species and tissue segment [124]. For example, the rat distal colon and human small intestine have a Young’s modulus as low as 0.3 kPa and 1.0 kPa, respectively [125]. The Young’s modulus of the porcine and human rectum can reach up to 1.8 and 5.2 MPa, respectively, and the tissues can elongate up to 2.1 and 1.6 their original length before failure, respectively [126].

Due to the high elasticity of the gastrointestinal tract, enteric microelectrodes may benefit from flexible substrates with greater compliance and decreased bending stiffness [127]. Ultra-soft microwire electrodes, for example, have Young’s modulus reportedly less than 1 MPa and may reduce the risk of intestinal injury [128]. Traditional microelectrodes such as monolithic silicon would be problematic due to their intrinsic stiffness, and would inevitably lead to increased cell death and pathophysiology [129]. Beyond the unique challenges of the gastrointestinal system, device characteristics such as electrical and insulative properties must also be considered. These material properties are discussed in detail by [130], and are summarized as: single-unit activity is better captured by low impedance and low surface area recording sites, with enough insulation to minimize parasitic capacitance.

### 4.2. Extrinsic Design Parameters

Extrinsic design parameters, such as probe geometry, electrode density, etc., can reduce the risk of disrupting gastrointestinal function and improve the signal-to-noise ratio of single-unit activity. First, enteric microelectrodes can increase flexibility with decreasing cross-sectional area, particularly probe thickness. For example, nanoelectronic thread electrodes are less than one-micron thick and “ultra-flexible”; the bending stiffness and mechanical interactions are on the order of cellular forces [131,132,133]. Ultrathin probes with a small cross-sectional area will be crucial to withstand the constant forces and movement within the gastrointestinal tract.

The macroscopic tissue movement in the gastrointestinal tract, and lack of nearby anchoring locations (i.e., skull, spine, etc.) pose additional challenges for enteric microelectrode design. The gastrointestinal environment will almost certainly demand a flexible tether between the anchored, transcutaneous connector and a recording platform [130]. The recording platform and enteric microelectrode must be anchored to the gastrointestinal wall without obstructing motility. Scaling up the mounting techniques from peripheral nerve interfaces, such as the spiral cuff [134] or locking-buckle cuff [135] are inappropriate, because they will prevent gastrointestinal distension and obstruct motility. Anchoring the recording platform with sutures through the serosa and muscular layers of the gastrointestinal wall will be less likely to obstruct the gastrointestinal tract and not directly disrupt barrier function [136,137,138].

Enteric microelectrodes should contain multiple recording sites along the length of the shank. To reach the myenteric plexus, the enteric microelectrode must penetrate the serosa and longitudinal muscle. Multiple recordings sites along the shank will allow a greater margin of error for probe depth and increase the likelihood of positioning a recording site near an enteric ganglion. The spacing between recording sites requires experimental optimization, and it will vary based on the insertion angle of the microelectrode. Importantly, multiple recording sites within the plexus layer will improve single-unit isolation [139]. Positioning additional recording sites in neighboring longitudinal or circular muscle layers may provide auxiliary physiological signals such as muscle action potentials or electrical slow wave activity. The additional recording sites and physiological signals could provide greater context for single-unit recordings or be used in signal processing techniques to increase the signal-to-noise ratio of single-unit recordings.

### 4.3. Implant Procedure

The implant procedure will greatly impact gastrointestinal physiology, and the procedure should be designed to reduce the risk of intestinal injury. A flexible microelectrode shank inserted into the gastrointestinal wall will be difficult to reliably position, and chronic macroscopic tissue motion will cause the electrode to drift over time, causing significant tissue damage [140,141]. To minimize the dimensions of tissue displacement relative to the probe, enteric microelectrodes should theoretically be implanted along the longitudinal axis, instead of the circumferential axis. However, this approach would be well-supported by experimental analysis.

Finally, enteric microelectrodes should be inserted at shallow angles relative to the serosa of the gastrointestinal wall. Microelectrodes should be designed to penetrate the longitudinal muscle layer without penetrating the submucosal layer. Piercing the epithelial layer or compromising barrier function will cause inflammation and sepsis [142]. Therefore the length of the microelectrodes and insertion angle should be designed specifically for the anatomy of the target species, because gastrointestinal dimensions scale across species [143].

## 5. Discussion

The available methods in enteric neuroscience are largely limited to excised tissue. While flat-sheet and whole-organ preparations are reliable tools to examine enteric neurophysiology, they are inadequate to study the interactions with the immune system, microbiota, extrinsic nervous system, etc. Anesthesia, on the other hand, modulates neurotransmission and impedes gastrointestinal motility, which confounds the interpretability of anesthetized in vivo recordings. Previously, we reported electrical activity from the enteric nervous system in anesthetized mouse, supported by simultaneous calcium imaging [144]. Although we observed increases in activity as expected with pharmacological stimulation and strong correlation with calcium activity, the source and robustness of the electrical activity remains disputed. This previous account demonstrates the challenges of anesthetized recordings, as well as the structural, physiological, and signal quality challenges in the gastrointestinal environment.

Single-unit recording capability from enteric neurons in awake animals has the potential to improve our understanding of the enteric nervous system, neurogastrointestinal function, and nutrition-mediated behavior. Single-unit resolution in awake animals will lead to computational models that better capture enteric neurophysiology which could guide future therapeutics [145,146]. Additionally, single-unit recordings pose great opportunities to synergize with advancements in other neurophysiology tools. Calcium imaging has been used reliably to monitor enteric neurons simultaneously in excised tissue [147,148] and anesthetized animals [144]. Furthermore, optogenetic stimulation and inhibition techniques have been adapted for enteric neurons [149], and have already been used to modulate motility in awake, freely-moving mice [150]. Additionally, neural microelectrodes designed for chronic, in vivo conditions have applications in electrical stimulation as an alternative to optogenetic stimulation.

## 6. Conclusions

In vivo electrophysiology in awake animals provides several opportunities and advantages over in vitro, ex vivo, and anesthetized in vivo recordings. Single-unit recordings from awake animals will require novel devices and methods to overcome the unique technical challenges posed by the gastrointestinal system. Importantly, single-unit recordings from awake animals have great potential to synergize with recent developments in optogenetics and in vivo imaging, but they will not completely replace traditional electrophysiology methods.

## Figures and Tables

**Table 1:** The effect of common anesthetic agents on various receptors of the enteric nervous system.

Neuron Species	Approximate Percentage	Affected Receptors	Inhibiting Anesthetic Agents	Potentiating Anesthetic Agents
Cholinergic	ChAT-positive neurons: ◦ 80% of myenteric neurons [29,34,35] ◦ 50% of submucosal neurons [29,34,35]	Neuronal nACh	Ketamine [36], pentobarbital [37], propofol [37], isoflurane [37,38], halothane [37,38], sevoflurane [37]	Urethane [39]
Purinergic	ATP-releasing neurons: ◦ 2–25% of myenteric neurons [28,40] ◦ 40–60% of submucosal neurons [28,40] ◦ Other: Enteric glia (P2X_7_) [41]	P2X_2_	Sevoflurane [42]	-
		P2X_3_	Pentobarbital [43]	-
		P2X_4_	-	Propofol [44]
		P2X_7_	-	Ketamine [45], propofol [45]
Serotinergic	5-HT-positive neurons: ◦ 2% of myenteric neurons [13]	5-HT_3_	Ketamine [46,47], pentobarbital [46], propofol [46]	Isoflurane [38,48], halothane [38,48]
Glutamatergic	NMDA-positive neurons: ◦ Almost all myenteric neurons [30] ◦ Almost all submucosal neurons [30]	NMDA	Ketamine [49], urethane [39], pentobarbital [50]	-
	AMPA-positive neurons: ◦ 30–60% of myenteric neurons [30] ◦ Almost all submucosal neurons [30]	AMPA	Urethane [39], pentobarbital [51], propofol [50]	-
	GABA_A_-positive neurons: ◦ 3–8% of myenteric and submucosal neurons [52,53]	GABA_A_	-	Ketamine [54], urethane [39], pentobarbital [55,56], propofol [54,57], isoflurane [54,58], halothane [54,58]
Glycinergic	Glycine-responsive: ◦ 57% of colonic myenteric neurons [31]	Glycine	-	Urethane [39], propofol [57], isoflurane [59], sevoflurane [59], halothane [59]

**Table 2:** The effect of common anesthetic agents on gastrointestinal motility during anesthesia.

Anesthetic Agent	Route of Administration	Gastric Emptying	Intestinal Transit
Ketamine	Injection	Unaffected [64,65]	Unaffected/slight decrease [64,65,66,67]
Urethane	Injection	Decrease [68,69,70,71]	Decrease [68,69]
Pentobarbital	Injection	Decrease [70]	Dose-dependent increase/decrease [66]
Propofol	Injection	Decrease [72,73]	Slight decrease [66,67]
Isoflurane	Inhalation	Decrease [74,75]	Decrease [62,76]
Sevoflurane	Inhalation	Decrease [77]	Decrease [77,78]
Halothane	Inhalation	Decrease [79]	Decrease [79,80,81]

**Table 3:** Key challenges to in vivo gastrointestinal neuro-electrophysiology.

Categories	Challenges
Structural	Large tissue displacements and no rigid structures on which to mount a device
Physiological	Ischemia and reperfusion injury Maintaining gastrointestinal homeostasis
Signal Quality	Electrical slow waves Smooth muscle action potentials Artifact due to tissue movement

**Table 4:** Enteric microelectrode design criteria for awake, single-unit recordings.

Design Criteria	Features
Material Properties	Low Young’s modulus High elasticity
Design Parameters	Low cross-sectional area Tethered recording platform Multiple recording sites along the length of the shank
Implant Procedure	Implant along longitudinal axis Shallow insertion angle Undisturbed submucosa and epithelial layer
